# Integrated Proteomic and Metabolomic prediction of Term Preeclampsia

**DOI:** 10.1038/s41598-017-15882-9

**Published:** 2017-11-23

**Authors:** Ray Bahado-Singh, Liona C. Poon, Ali Yilmaz, Argyro Syngelaki, Onur Turkoglu, Praveen Kumar, Joseph Kirma, Matthew Allos, Veronica Accurti, Jiansheng Li, Peng Zhao, Stewart F. Graham, David R. Cool, Kypros Nicolaides

**Affiliations:** 1 Department of Obstetrics and Gynecology, Beaumont Health, Royal Oak, MI USA; 2 Harris Birthright Research Centre for Fetal Medicine, King’s College Hospital, London, UK; 3 Department of Obstetrics and Gynecology, The Chinese University of Hong Kong, Shatin, NT, Hong Kong SAR, China; 4 Oakland University, Rochester Hills, MI USA; 5 Henan University of Chinese Medicine, Zhengzhou, Henan China; 6 Department of Pharmacology & Toxicology, Wright State University, Dayton, OH USA; 7 Department of Obstetrics & Gynecology, Wright State University, Dayton, OH USA

## Abstract

Term preeclampsia (tPE), ≥37 weeks, is the most common form of PE and the most difficult to predict. Little is known about its pathogenesis. This study aims to elucidate the pathogenesis and assess early prediction of tPE using serial integrated metabolomic and proteomic systems biology approaches. Serial first- (11–14 weeks) and third-trimester (30–34 weeks) serum samples were analyzed using targeted metabolomic (^1^H NMR and DI-LC-MS/MS) and proteomic (MALDI-TOF/TOF-MS) platforms. We analyzed 35 tPE cases and 63 controls. Serial first- (sphingomyelin C18:1 and urea) and third-trimester (hexose and citrate) metabolite screening predicted tPE with an area under the receiver operating characteristic curve (AUC) (95% CI) = 0.817 (0.732–0.902) and a sensitivity of 81.6% and specificity of 71.0%. Serial first [TATA box binding protein-associated factor (TBP)] and third-trimester [Testis-expressed sequence 15 protein (TEX15)] protein biomarkers highly accurately predicted tPE with an AUC (95% CI) of 0.987 (0.961–1.000), sensitivity 100% and specificity 98.4%. Integrated pathway over-representation analysis combining metabolomic and proteomic data revealed significant alterations in signal transduction, G protein coupled receptors, serotonin and glycosaminoglycan metabolisms among others. This is the first report of serial integrated and combined metabolomic and proteomic analysis of tPE. High predictive accuracy and potentially important pathogenic information were achieved.

## Introduction

Preeclampsia (PE) is a common obstetric disorder seen in 3–5% of pregnancies in developed countries^1^. Substantial evidence exists, based on placental histology^2^, maternal demographic characteristics^3^, biochemical^4^ and uterine artery Doppler^5^ data, suggesting that PE might be two distinct disorders. These include an early-onset variety requiring delivery at <34 weeks and a late-onset variety requiring delivery at ≥34 weeks. Alternatively, the two disorders could represent extremes of a disease spectrum^6^. Early-onset PE is the more severe form of the disorder while late-onset PE is 3–7 times more common^3^. However, late-onset PE (usually classified as mild-PE) is not a benign disorder and is associated with increased short-term risks such as fetal growth restriction, perinatal death and morbidities^3^ and long-term maternal cardiovascular disease such as ischemic artery disease, congestive heart failure, stroke, thromboembolism, type 2 diabetes^7^ and cardiomyopathy^8^. Recently published data suggest that unlike aspirin prophylaxis, metformin could be effective for late or mild PE prevention^9^. However, to conduct optimal prophylaxis trials, accurate screening markers for PE are needed. A recent recommendation from United States Preventive Services Task Force (USPSTF) also indicated that there is a substantial net benefit of screening for PE in pregnant women^10^. USPSTF also opined that there is inadequate evidence to support the effectiveness of current risk assessment tools beyond blood pressure measurement and that further research was needed to validate the clinical utility of other risk prediction models^10,11^. Highly accurate screening tests are required for early prediction and pharmaceutical prophylaxis of different types of PE.

Term PE (tPE), requiring delivery at ≥37 weeks, is the most common subgroup of PE, accounting for as much as 60% of PE overall and appears to be the most difficult type to predict. Given its frequency and obstetric and long-term health consequences, there has been an increase in research in the prediction of late-onset including term PE. A recent series of studies by the group at Kings College Hospital, London, reported on first, second and third trimester algorithms for PE prediction. They utilized uterine artery Doppler^11^, various biochemical markers^12–14^, mean arterial blood pressure (MAP)^15^, and maternal characteristics^16^ individually or in combination for PE prediction. Several conclusions were drawn from these series of studies. Firstly, the predictive accuracy declined as the gestational age at clinical onset increased. Early PE was more accurately predicted than intermediate (delivered at 34–37 weeks) and tPE. Term PE had the lowest predictive accuracy with detection rates of between 37% and 73% at a false positive rate (FPR) of 10%^12–15^. Thus, the later the screening is performed, i.e. shorter the interval between screening and disease onset, the higher the diagnostic accuracy that was found. Finally, combining markers from more than one trimester i.e. using serial measurements enhanced the predictive accuracy.

Metabolomics, focuses on the high-throughput identification and quantification of small molecule metabolites and assesses their interactions within biological networks^17^. Metabolomics rapidly reflects cellular perturbations and is a sensitive identifier of cell phenotype. Our group has previously utilized metabolomics to identify biomarkers for the first trimester prediction of late-PE^18^. Proteomics, the large-scale study of proteins in an organism, is a more familiar and widely used systems-biology approach. It deepens insights into cellular pathways and regulatory mechanisms and is extensively used for the discovery of biomarkers and identification of new therapeutic targets. The integration of proteomics and metabolomics, has been proposed to further elucidate disease pathogenesis and to improve biomarker prediction in cancer research^19–21^. In this study, we evaluated the use of integrated metabolomic and proteomic analysis to more deeply interrogate the mechanisms of and to develop predictive biomarkers for tPE.

## Results

A total of 35 cases with subsequent tPE and 63 maternal age-matched controls were included in the study. The maternal demographics and clinical variables of tPE cases and controls are presented in Table 1. Clinical variables including MAP, body mass index (BMI), and history of PE were recorded on both first and third trimester visits, however none of them achieved statistical significance for tPE prediction in the models which considered omics data.

**Table 1:** Comparison of demographics and clinical assessments: Preeclampsia cases vs Controls.

	Mean (SD)		p-value
	Preeclampsia	Controls	
Number	35	63	—
Age	32.3 (5.5)	32.6 (5.8)	0.83^+^
Parity			
• Nulliparous	18	37	0.43*
• Multiparous	17	26	
Race			
• White (n)	15	39	0.13^
• Black (n)	16	19	
• Asian (n)	4	3	
• Mixed (n)	0	2	
BMI (11^+0^–13^+6^ wks)	27.1 (6.4)	25.6 (4.2)	0.14^+^
BMI (32^+0^–33^+6^ wks)	29.0 (7.1)	29.4 (5.8)	0.79^+^
MAP mom (11^+0^–13^+6^ wks)	1.013 (0.071)	1.032 (0.090)	0.28^+^
MAP mom (32^+0^–33^+6^ wks)	1.015 (0.080)	1.027 (0.093)	0.54^+^
Previous PE, N (%)			
• Multipara-PE history	4 (12%)	5 (9%)	0.21^#^
• Multipara-no PE history	13 (37%)	21 (33%)	
• Nullipara	18 (51%)	37 (58%)	
FH PE-Mother, N (%)	1	0	—

BMI: Body mass index, MAP: Mean arterial pressure, PGFL: Placenta growth factor, FH PE-Mother: Family history of Preeclampsia - Mother. ^+^t-test.

*Chi square test.

^Kruskal Wallis.

^#^Fisher’s Exact test.

Targeted metabolomics approach identified 181 metabolites using Direct Injection Liquid Chromatography coupled with mass spectrometry (DI-LC-MS/MS) and 47 with Nuclear Magnetic Resonance (^1^H NMR) spectroscopy for both first and third trimester specimens. There were 20 overlapping metabolites between the two platforms and duplicate measures were removed from the analysis. The mean (SD) concentrations of first (Supplementary Table S1) and third (Supplementary Table S2) trimester metabolites (μM) acquired using DI-LC-MS/MS and ^1^H NMR, were compared between cases and controls. The relative change in metabolites in the future tPE group vs. controls, fold-change and p-values are presented. For the first trimester: glucose, putrescine, PCaaC40:6, urea and dimethyl sulfone and for the third trimester: serotonin, t4-OH-proline, hexose, acetic acid and dimethyl sulfone were significantly altered in tPE after controlling for multiple comparisons (p-value < 0.05). Combined NMR and DI-LC-MS/MS metabolomic analysis of first trimester metabolites did not yield statistically significant separation between tPE and controls. The permutation testing using 2000 repeats yielded a p-value = 0.33 indicating that the observed separation between groups could be due to chance. Similarly, third trimester metabolites by themselves did not significantly separate tPE and controls. Permutation testing using 2000 repeats yielded a p-value = 0.44. The failure of metabolites *in individual* trimester to significantly separate the tPE group from controls was likely due to insufficient study power.

We also analyzed the samples using a proteomics platform and 183 features were identified in the serum proteome of first and third trimester samples using matrix assisted laser desorption ionization time of flight MS (MALDI-TOF/TOF-MS). Among these ions of interest, 41 were considered to be of significant interest, p-value < 0.3 and potentially useful for distinguishing cases from controls (Supplemental Table S3). Three features (594 *m/z*, 650 *m/z*, 636 *m/z*) were significantly upregulated in tPE cases (p < 0.05). Partial Least Squares Discriminant Analysis (PLS-DA) and Variable Importance in Projection (VIP) plots for first trimester tPE prediction were performed. Permutation testing (n = 2000 repeats) did not demonstrate a statistically significant separation of the groups based on first trimester proteomic analysis (p = 0.64). The chemically unidentified feature - m/z of 594, was ranked as first while 60 S ribosomal protein L41 (RL41), tumor necrosis factor-alpha (TNF-α), sperm-associated antigen 11B (SG11B), and immunglobulin light chain variable region (Q8TE63) were ranked 2 through 6 in terms of separation power in the PLS-DA models based on VIP plot. For the third trimester specimens we identified 75 features as potentially useful (P < 0.3) for discriminating tPE and controls with 37 of them demonstrating statistically significant changes in concentrations (p < 0.05) between the tPE and control groups (Supplementary Table S4). The PLS-DA plot (Supplemental Fig. S1A) shows significant separation between third trimester cases and controls: permutation testing (2000 repeats) was statistically significant (P < 0.0005). Based on the VIP plot, Putative protein FAM86JP (F86JP) appeared to be the top ranking protein for third trimester prediction of tPE (Supplemental Fig. S1B).

Multivariate logistic regression analysis was performed to construct biomarker models for tPE prediction. Table 2 presents the first trimester-only predictive models for the subsequent development of tPE. First trimester maternal demographics and clinical factors by themselves did not significantly predict subsequent tPE development. Lack of significance is likely due to the small sample size, in view of prior large scale studies and clinical guidelines supporting the use of such clinical/demographic features for PE prediction. At the very least, the findings suggest that demographic and clinical markers are not powerful predictors of PE compared to omics markers and achieve significance only in large data sets. Based on metabolomic only data, a combination of putrescine, urea and carnitine concentrations produced the best first trimester prediction model with an Areas under the Receiver Operating Characteristic curve (AUROC or AUC) (95% CI) = 0.701 (0.589–0.814) and sensitivity = 72.7% and specificity = 57.4%. The first trimester-only proteomics model using TNF-α, RPL41, ATP synthase subunit epsilon (ATP5E) and TATA-box-binding protein (TBP) was the best first trimester predictor: AUC (95% CI) = 0.694 (0.578–0.811) with a sensitivity = 66.7% and specificity = 74.1%. The combined metabolomics and proteomics first-trimester model consisting of TNF-α, RPL41, ATP5E, TBP, putrescine, urea and carnitine yielded an AUC (95% CI) = 0.745 (0.638–0.852) with sensitivity = 78.8% and specificity = 64.8% (Table 2).

**Table 2:** Proteomics and metabolomics models^*^ in *first trimester* prediction of term preeclampsia.

Model	Predictors	AUC (95% CI)	Sensitivity % (95% CI)	Specificity % (95% CI)
Maternal factors	Parity, MAP (12 wks), BMI (12 wks)	0.565 (0.442–0.688)	62.9% (62.9–78.9)	50.8% (38.4–63.1)
Metabolites	Putrescine, Urea, Carnitine	0.701 (0.589–0.814)	72.7% (72.7–87.9)	57.4% (44.2–70.6)
Peptides	TNF-α, RPL41, ATP5E, TBP	0.694 (0.578–0.811)	66.7% (66.7–82.8)	74.1% (62.4–85.8)
Metabolites + peptides	TNF-α, RPL41, ATP5E, TBP, Putrescine, Urea, Carnitine	0.745 (0.638–0.852)	78.8% (78.8–92.7)	64.8% (52.1–77.6)

TBP: TATA box binding protein - associated factor; RPL41: 60S ribosomal protein L41; TNF-α: Tumor necrosis factor alpha (fragment); ATP5E: ATP synthase subunit epsilon; MAP: Mean arterial pressure; BMI: Body mass index.

*Based on logistic regression analysis, threshold values are presented in the supplementary information (Supplementary Table S6).

Third trimester-only predictive models are exhibited in Table 3. The combination of methylhistidine, serotonin, citrate, hexose and propylene glycol produced the best third trimester metabolite model with an AUC (95% CI) = 0.761 (0.648–0.875), sensitivity = 74.2% and specificity = 72.3%. Human Leukocyte Antigen D related Beta-1 (HLA-DR B1) combined with GTP binding protein-3 (GTPBP3), had an AUC = 0.985 (0.956–1.000), with sensitivity and specificity equal to 100% and 98.4%, respectively. We also evaluated an alternate predictive third trimester peptide model, which included Testis-expressed sequence 15 protein (TEX15) and Stathmin 3 (SCG10). This combination achieved an AUC (95% CI) = 0.937 (0.862–1.000) with sensitivity and specificity equal to 94.3% and 98.4%, respectively. Maternal demographics and clinical predictors again did not improve the performance of third trimester omics models alone.

**Table 3:** Proteomics and metabolomics models* in *third trimester* prediction of term preeclampsia.

Model	Predictors	AUC (95% CI)	Sensitivity % (95% CI)	Specificity % (95% CI)
Maternal factors	Parity, MAP (32 wks), BMI (32 wks)	0.525 (0.405–0.644)	54.3% (54.3–70.8)	52.4% (40.0–64.7)
Metabolites	Methylhistidine, Serotonin, Citrate, Hexose, Propylene glycol	0.761 (0.648–0.875)	74.2% (74.2–89.6)	72.3% (59.6–85.1)
Peptides (top performing model)	GTPBP3, HLA-DR β-1 MHC	0.985 (0.956–1.000)	100% (100–100)	98.4% (95.3–100)
Peptides (2^nd^ model)	TEX15, SCG10	0.937 (0.862–1.000)	94.3% (94.3–100)	98.4% (95.3–100)
Peptides (top model) + demographics	GTPBP3, HLA-DR β-1 MHC, MAP (32 wks), BMI (32 wks)	0.941 (0.879–1.000)	91.4% (91.4–100)	96.8% (92.5–100)

HLA-DR β-1 MHC: Human Leukocyte Antigen - antigen D Related Beta 1 major histocompatibility complex; GTPBP3: GTP binding protein 3; TEX15: Testis-expressed sequence 15 protein; SCG10: Stathmin 3; MAP: Mean arterial pressure, BMI: Body mass index.

*Based on logistic regression analysis, threshold values are presented in the supplementary information (Supplementary Table S6).

In addition to individual first and third trimester models, we developed serial (integrated) models which combined biomarkers from the first and third trimesters (Table 4). Serial modeling improved metabolomic prediction of tPE. First (urea and SM C18:1) and third trimester metabolites (citrate and hexose) when combined yielded an AUC (95% CI) = 0.817 (0.732–0.902) with sensitivity = 81.6% and specificity = 71.0%. The serial integrated peptide model which included the first trimester protein, TBP and the third trimester protein TEX15 had an AUC (95% CI) = 0.987 (0.961–1.000) with sensitivity = 100% and specificity = 98.4%.

**Table 4:** Proteomics and metabolomics models* in *serial* integrated (first and third trimester) prediction of term preeclampsia.

Model	Predictors	AUC (95% CI)	Sensitivity % (95% CI)	Specificity % (95% CI)
Maternal factors	MAP (32 wks), MAP (12 wks), BMI (12 wks), BMI (32 wks),	0.582 (0.460–0.705)	48.6% (48.6–65.1)	71.4 (60.3–82.6)
Peptides (top model)	TEX15 (3^rd^ tr), TBP (1^st^ tr)	0.987 (0.961–1.000)	100% (100–100)	98.4% (95.3–100)
Peptides (2^nd^ model)	GTPBP3 (3^rd^ tr), RPL41 (1^st^ tr)	0.983 (0.953–1.000)	97.1% (97.1–100)	98.4% (95.3-100)
Peptides + demographics	GTPBP3 (3^rd^ tr), SCG10 (3^rd^ tr), ATP5E (1^st^ tr), BMI32 wks, MAP32 wks, MAP12 wks	0.977 (0.949–1.000)	97.1% (97.1–100)	90.5% (83.2–97.7)
Metabolites	Urea (1^st^), SM C18:1 (1^st^), Citrate (3^rd^), Hexose (3^rd^),	0.817 (0.732–0.902)	81.6% (81.6–93.9)	71.0% (60.3–81.7)
Metabolites + demographics	Urea (1^st^), Hexose (3^rd^), SM C18:1 (1^st^), Citrate (3^rd^), MAP(32 wks), BMI (12 wks)	0.805 (0.717–0.894)	84.2% (84.2–95.8)	71.0% (60.3–81.7)

TEX15: Testis-expressed sequence 15 protein; TBP: TATA box binding protein - associated factor; GTPBP3: GTP binding protein 3; RPL41: 60S ribosomal protein L41; SCG10: Stathmin 3; ATP5E: ATP synthase subunit epsilon; MAP: Mean arterial pressure; BMI: Body mass index.

*Based on logistic regression analysis, threshold values are presented in the supplementary information (Supplementary Table S6).

### Pathway analyses

**Figure 1:**
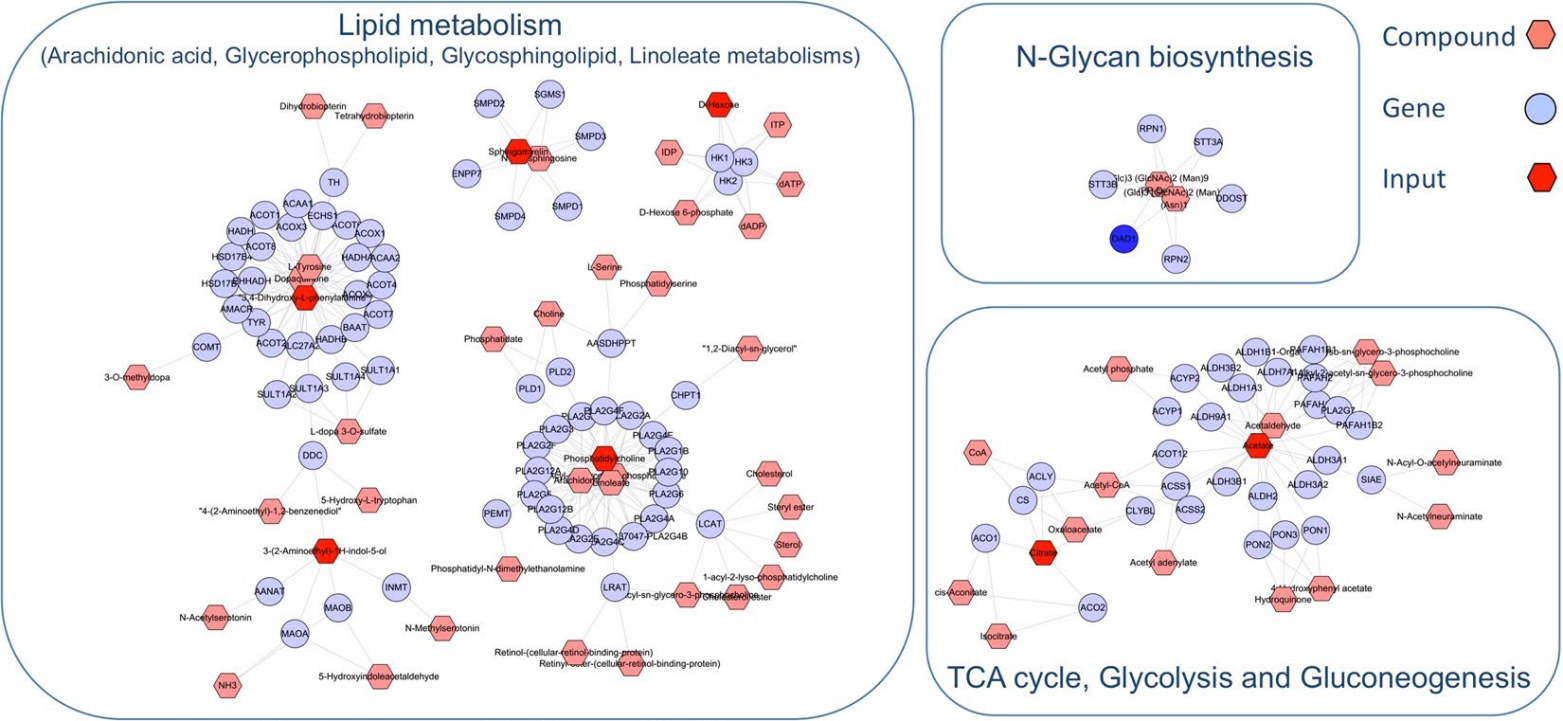
Correlation network of the third trimester metabolites and proteins in future term preeclampsia.

Pathway topology analysis using third trimester metabolites revealed significant differences in multiple metabolic pathways between controls and future tPE (Supplementary Table S5). Pathways included but were not limited to pyruvate, amino sugar and nucleotide sugar, glyoxylate and dicarboxylate, glycerophospholipid metabolisms and citrate cycle (TCA cycle). In addition to the metabolomics pathway topology analysis, our integrated pathway over-representation analysis limited to third trimester metabolomic and proteomic data (obtained prior to disease development) identified markedly altered biological pathways in cases destined to develop tPE. The top ranking over-represented pathways include: signal transduction, G protein-coupled receptor (GCPR), serotonin and glycosaminoglycan metabolisms. The significant pathways with associated p-values, number of overlapping genes and metabolites are presented in Table 5. Additionally, we constructed a correlation network diagram using Metscape^22^. This approach generates a comprehensive picture of latent relationships between metabolites and proteins (Fig. 1). Metabolite–protein correlation networks consisted of lipid metabolism (arachidonic acid, glycerophospholipid, glycosphingolipid, linoleate metabolisms) in addition to N-glycan biosynthesis, glycolysis, gluconeogenesis and the TCA cycle. We were unable to perform an integrated pathway analysis for first trimester metabolomics and proteomics data due to low numbers of confidently identified peptides in UniProt Knowledgebase based on the results generated from the MASCOT peptide mass fingerprinting (PMF) search engine.

## Discussion

Term PE (≥37 weeks) is common but more difficult to predict. Late-onset PE, the majority of which are tPE cases is associated with micro-vascular disorders such as hypertension, diabetes and obesity decades after the obstetric manifestations^23^. A combined serial first and third trimester (integrated) metabolomics model exhibited good predictive accuracy for tPE: AUC (95% CI) = 0.817 (0.732–0.902). The corresponding serial integrated first and third trimester proteomic model was highly predictive of tPE: AUC (95% CI) = 0.987 (0.961–1.000). Using a combined proteomics and metabolomics approach, improved first trimester prediction of tPE. However, there was no significant improvement in performance over third trimester proteomic markers alone versus when metabolomics and proteomic markers were i) combined in the third trimester or ii) when serially integrated i.e. first- and third trimester proteomics and metabolomics markers were combined for tPE prediction. This was due to the high diagnostic accuracy of the proteomic markers by themselves. Additionally, maternal risk factors either in the first or third trimester, did not present any significant difference between tPE cases and controls. Further, combining maternal risk factors with metabolomics and proteomics models did not further improve the predictive accuracy.

We also combined proteomic and metabolomics pathway analysis and this yielded important insights into the pathogenesis of tPE including changes in G-protein coupled receptors (GPCR) signaling, serotonin and mucopolysaccharide metabolism. All of these mechanisms have been linked to cardiovascular and hypertensive disorders and can be plausibly linked to PE. These will be discussed further. To our knowledge, the combination of proteomic and metabolomics data for understanding the mechanism of PE has not been previously reported. While several other groups have evaluated metabolomic^24,25^ and proteomic markers^26,27^ for PE prediction, there are no prior publications that combine the two omics or integrate first and third –trimester screening as reported here. Further, we could find no other omics study that was limited to term PE.

Individually metabolomics and proteomics provided important information about the mechanism of tPE. The concentrations of dimethyl-sulfone, or methylsulfonylmethane (MSM) remained significantly lower in tPE cases compared to controls across both trimesters. MSM is naturally occurring organic sulfur that is known as a potent antioxidant/anti-inflammatory compound^28^, which has been found to improve various metabolic diseases by decreasing oxidative stress^29,30^. Oxidative stress is well known to be an important metabolic feature of PE. In third trimester peptide model, increased levels of HLA-DR B1 and GTPBP3 contributed to the prediction of tPE. Increased levels of HLA-DR B1 gene expression^31–33^ and soluble HLA-DR B1^34^ were previously reported to be associated with PE. In a small study, increased levels of HLA-DR B1 gene expression in early third trimester were reported to precede the onset of PE^35^. Additionally, current data report an inconsistent association between GTP3B polymorphisms and essential hypertension and PE^36,37^ which requires further investigation.

Integrated pathway over-representation analysis performed using IMPALA revealed significantly altered pathways in third trimester pregnant women who later went on to develop tPE (Table 5). G-protein coupled receptors (GPCR) are involved in the initiation of a diverse range of important signaling pathways that control diverse functions such as heart rate, contractility, vascular tone, and blood volume for example. Important cardiovascular disorders such as diabetes and hypertension are associated with abnormal GPCR signaling. Reductions of endogenous ligands of various GPCRs such as AT2 (angiotensin 2) and relaxin for example, are reportedly decreased in preeclamptic pregnancies^38^. Further, the use of GPCR agonists and antagonists has been extensively reported as therapeutic targets in PE^38^. We have previously published metabolomic evidence of the likely role of insulin resistance in the pathogenesis of late-onset PE^18^. Subsequent evidence suggested that metformin might be an effective prophylaxis against (primarily) late-PE^9^. Metformin has been reported to disrupt the crosstalk between insulin and GPCR signaling pathways in human pancreas^39^.

**Table 5:** Over-representation pathway analysis of term preeclampsia using integrated third trimester Proteomics and Metabolomics.

Pathway	No. of overlapping genes	No. of genes in pathway	*p*-value	*q-*value^*^	No. of overlapping metabolites
Signal Transduction	6	2496 (2538)	0.000	0.337	5
Signaling by GPCR	4	1298 (1310)	0.001	0.337	4
Serotonin Receptor 4–6–7 and NR3C Signaling	1	19 (19)	0.002	0.337	1
Neurotransmitter Clearance In The Synaptic Cleft	0	0 (0)	0.002	0.943	2
Excitatory Neural Signaling Through 5-HTR 4 and Serotonin	1	8 (8)	0.003	0.337	1
Excitatory Neural Signaling Through 5-HTR 6 and Serotonin	1	8 (8)	0.003	0.337	1
Excitatory Neural Signaling Through 5-HTR 7 and Serotonin	1	8 (8)	0.003	0.337	1
Amine ligand-binding receptors	0	43 (43)	0.003	0.337	2
Post-translational modification: synthesis of GPI-anchored proteins	2	93 (93)	0.004	0.338	1
Glycosaminoglycan metabolism	0	0 (0)	0.005	0.943	2
GPCR ligand binding	0	0 (0)	0.005	0.943	3

*q-value: The False Discovery Rate which results from correcting the P-values for multiple testing using the method set out by Benjamini and Hochberg.

Five hydroxytryptamine (5-HT, serotonin) is extensively involved in cardiovascular responses in normal and disease states. These responses include heart rate, vasodilation, vasoconstriction and hypotension or hypertension. The effect of 5-HT in a particular vascular bed depends on the type of 5-hydroxytryptamine receptors (5HTR) present. Increased concentrations of serotonin and alterations in 5HTR have been reported in PE^40^, consistent with it’s major role in controlling blood pressure. The main source for circulating serotonin is platelets. Platelet aggregation is an important feature of PE and causes an increase in serotonin release. Serotonin acting directly on vascular beds and especially via excitatory neural signaling through 5-HTR, is known to play a role in the stimulation of vascular smooth muscle and endothelial cells^41^. We found an alteration of serotonin pathways in the third trimester, before the onset of clinical tPE, possibly consistent with a causative role in PE.

Glycosaminoglycans (GAG, mucopolysaccharides) are long unbranched polysaccharides consisting of repeated disaccharide units. GAGs are localized both in the extracellular matrix and on the cell surface and play a role in cell survival, migration and also in angiogenesis. The placenta contains large amounts of GAGs that are located predominantly in the intervillus space. Alterations in extracellular matrix (including GAG) with increase in heparan sulfate, dermatan sulfate and heparanase activity have been reported in the placentas of patients with PE^42^. Those authors posited that dermatan sulfate might be involved in trophoblast invasion. Vascular endothelial growth factor required for placental angiogenesis is known to be regulated by heparan sulfate. Most interestingly, heparin sulfate can bind soluble fms-like tyrosine kinase-1, known to be elevated in PE, and might thus enable this receptor to bind to placental blood vessels and inhibit angiogenesis.

We also integrated the third trimester proteomics and metabolomics data into a network map that includes peptides, expressed genes and metabolites. Lipid metabolism, including arachidonic acid, glycerophospholipid, glycerosphingolipid and linoleate metabolisms, were found to be the most perturbed biologic pathways in patients with future tPE. First trimester lipid metabolism perturbations are known to occur in patients who subsequently developed late-onset PE^43,44^. In addition to the lipid metabolism, energy metabolism including the TCA cycle, glycolysis and gluconeogenesis were significantly perturbed in the third trimester. Our results from the third trimester metabolomics pathway topology analysis were consistent with the integrated network analysis revealing perturbed energy and lipid metabolisms in future tPE (Supplementary Table S5). In concordance with our current results, we previously reported alterations in cellular energy metabolism in late-onset PE^18^. We found n-glycan biosynthesis to be an overrepresented pathway in our integrated analysis. Glycosylation, i.e. the enzymatic addition of N-glycan’s to proteins and lipids, determines many properties of glycoproteins including their conformation, solubility, and antigenicity^45^. Previous reports support altered glycosylation in plasma^46^ and placental tissue proteins^47,48^ in PE. Further investigation of the role of glycosylation in PE, especially in tPE, is clearly warranted.

Overall, demographic and clinical risk factors were not found to be strong tPE predictors in this study. This is possibly due to our relatively small sample size as compared to the larger studies that identified demographic factors as significant predictors of PE. Our findings do not mean that demographic factors are not predictors of PE however. Rather, they suggest however that clinical/demographic factors are modest risk factors in terms of their effect sizes and thus they require much larger study numbers to achieve statistical significance as compared to omics markers. Omics markers have higher predictive abilities and thus still achieve statistical significance in a smaller study population. Further, it should be borne in mind that omics provide cellular markers of these same demographic and clinical factors such as obesity/BMI. This reduces the incremental impact of the demographic and clinical markers to the predictive equations since to a large degree they are already accounted for in the omics markers. Finally, clinical markers such as mean arterial pressure^49^ and uterine artery Doppler velocimetry^11^ are known to perform significantly better for the prediction of early- compared to late-onset or term PE which is the subject of the current study.

This study is not without limitations. First, our sample size was modest making it difficult to evaluate the performance of the models in a separate independent validation sub-group. Despite the relatively small sample size of our study, we have ensured the generalizability of our results by performing stringent cross-validation such as permutation tests (2000 repetitions) and 10-fold CV. Using a larger numbers of cases and controls may potentially lead to the identification of additional significant metabolites and proteins and additional biochemical pathways involved in this complex disorder. Further, a number of additional peptides have not yet been definitively identified at the time of the manuscript submission. Determining the identity of these peptides is expected both to improve existing predictive model performance and enhance our mechanistic understanding of late-PE.

Overall, this novel study presents the integration of metabolomics and proteomics for the study of tPE. First-only, third-only and the serial combination of metabolite and peptides significantly predicted tPE. Third trimester and serial models produced the strongest tPE predictive algorithms. Importantly, by integrating metabolomics and proteomics, we have highlighted biochemical pathways plausibly associated with the underlying pathogenesis of tPE. Finally, a more precise understanding of PE mechanism as generated by this approach, and could help to increase the chances of the identification of novel pharmacological therapies for the prevention and treatment of PE. While our findings are strong and promising, additional studies encompassing larger sample cohorts and different populations appear warranted which will allow us to validate our findings as reported herein.

## Materials and Methods

### Study population and sample collection

This case-control study is part of an on-going first and third trimester screening of the general obstetric population for prediction of obstetric and fetal complications. The study was reviewed and approved on 03–14–2003 by the UK Research Ethics Committee (Project #02-03-033). Informed consent forms were obtained from all the study participants and all methods were performed in accordance with relevant guidelines and regulations. The details of such protocols have been previously described^9–15^. Following written consent, women were recruited at the time of first trimester aneuploidy screening, between 11^+0^–13^+6^ weeks’ gestation. Maternal characteristics and medical history were recorded for each participant. Patients with multiple pregnancies and known or suspected major structural, chromosomal or genetic abnormalities were excluded from the study population. Gestational age was confirmed by ultrasound measurement of fetal crown-rump length (CRL). The second study visit was held between 32^+0^–33^+6^ weeks’ gestation during the provision routine prenatal care. First and later third trimester maternal blood samples were collected and incubated for 30 minutes at room temperature to allow clotting and subsequently centrifuged at 3000 rpm for 10 minutes to separate the serum from clots. Serum samples were aliquoted into 0.5 mL quantities and stored at −80 °C within 1 hour of the maternal blood collection. Term PE was defined as proposed by the International Society for the Study of Hypertension in Pregnancy (14) with systolic blood pressure ≥140 or diastolic ≥90 mm Hg on two or more occasions 4 hours apart after 37 weeks of gestation, in previously normotensive women. Proteinuria was defined as a total of 300 mg in a 24-hour urine collection or two readings of at least 2^+^ of proteinuria in the absence of a 24-hour urine collection, which must also have been present in addition to the hypertension. Subsequently, controls matched for gestational age at delivery i.e. ≥37 weeks were chosen randomly. Only phenotypically normal cases were included in the study overall. For controls, only cases with appropriate birth weight for gestational age and who did not develop any hypertensive disorder were considered. During the selection of age-matched controls, laboratory authors, not clinically involved in the care of the patient were blinded to the other confounding factors of preeclampsia, including race, parity, BMI, MAP and history of preeclampsia. Cases with insufficient sample volume to do both proteomic and metabolomics analyses in both the first and third trimester, those in which all the relevant metabolites or peptides used for the statistical analysis could not be measured in the samples or samples with evidence of hemolysis were excluded from further statistical or bioinformatics analysis and were therefore not reported on.

### Metabolomic analysis

#### NMR based metabolomic analysis

NMR spectra was acquired as described by Mercier *et al*.^50^. In brief, ^1^H-NMR spectra were recorded at 300 K on a 600-MHz Avance III HD Bruker spectrometer (Bruker Biospin Inc, Billerica, MA) equipped with a triple resonance inverse detection TCI cryoprobe operating at 600.13 MHz. Sample preparation and data acquisition for NMR based metabolomics are detailed in the Supplementary methods section.

#### DI-LC-MS/MS based metabolomic analysis

The Absolute IDQ p180 kit (Biocrates Life Sciences AG, Innsbruk, Austria) with a TQ-S mass spectrometer coupled to an Acquity I Class ultra-pressure liquid chromatography (UPLC) system (Waters Technologies Corporation, Milford, MA, USA) was used to perform targeted analysis of metabolites which included amino acids, acylcarnitines, biogenic amines, glycerophospholipids, sphingolipids, and sugars. Sample preparation and data acquisition for DI-LC-MS/MS are also detailed in the Supplementary methods section.

### Proteomic analysis

Serum samples were diluted (1:5) with 20% acetonitrile in water, then centrifuged at 3,000 *g* for 30 minutes using an Amicon Ultra-4 spin column (50 kDa cut-off) to remove the majority (≥80%) of albumin and other highly abundant high-molecular weight proteins. Twenty µl of the serum filtrate was loaded onto a preconditioned ZipTip C18 tip-microcolumn (preconditioned using 20 μl of methanol and washed with 20 μl of 0.1% TFA solution) by continually passing it through the tip (x10). Following this step the column was washed with 0.1% TFA (x5) and subsequently eluted with 90% ACN in 0.1% TFA. The eluate (1 μl) was mixed with 1 μl of CHCA MALDI matrix in 50% ACN and 0.1% TFA. 1 μl of the mixture was spotted onto a Bruker MTP384 ground steel target plate. MALDI-TOF spectra were acquired using a Bruker Autoflex III (Bruker, Daltonics). Each spectrum is the sum of 10,000 laser shots collected randomly at a frequency of 100 Hz. The target mass range was 700–10,000 Da applying both MS reflector in positive ion mode and linear positive ion mode. Data was analyzed using FlexAnalysis v2.0 (Bruker Daltonics). Internal calibration was based on the five standard peptides (PepMixII). For sequencing, protein parent ions were captured in LIFT-MS mode followed by fragmentation using LIFT-MS/MS. The parent and fragments were collected and used to search for identities in the MASCOT peptide mass fingerprinting (PMF) search engine (http://www.matrixscience.com). Search criteria included: 0.5–1.2 Da mass error tolerance, two missed cleavage sites permitted, methionine oxidation as variable modification and carbamidomethyl (cysteine) as fixed modification. The database employed was NCBInr 20060712. All spectra were loaded into ClinProTools v2.0 software were baseline-subtracted, smoothed, normalized and realigned. Any spectra, which could not be realigned in this process, were not included for further analysis. Finally, peak intensities were normalized to Total Ion Current (TIC)^51^.

### Statistical analysis

To examine the distribution of the clinical variables: age, parity, race, BMI, MAP, prior and also family history of PE were analyzed using the Kolmogorov-Smirnov test. Metaboanalyst v3.0 and IBM SPSS 22.0 programs were used to perform all the statistical analyses. For metabolomic and proteomic data analyses both univariate and multivariate statistical analyses were applied. Mean (SD) metabolite concentrations and peptide abundances in cases and controls were compared using a two-tailed t-test. The Mann Whitney U test was performed on variables with non-normal distributions. The data were normalized to the sum and auto-scaled prior to multivariate analyses. Using Metaboanalyst v3.0, Principal Component Analysis (PCA) and PLS-DA were performed to identify distinct metabolite and peptide patterns. Permutation testing (2000 repeats) was performed to assess whether the observed separation achieved by PLS-DA was due to chance^52^. Additionally, VIP plots were generated to rank metabolites and peptides based on their ability to predict tPE cases.

Logistic regression analysis was used to generate the optimal predictive models for tPE. Independent clinical variables and potential confounders were considered in each of the prediction models. A k-fold cross-validation (CV) technique was employed to ensure that the logistic regression models were robust^53^. Further variable/predictor selection methods including LASSO (Least Absolute Shrinkage and Selection Operator)^54^ and stepwise variable selection were utilized to optimize model components^55^ with 10-fold CV. The threshold used for inclusion of metabolites, peptides or other clinical predictors required that the variable be selected >8 times to be included in the logistic equations for tPE prediction^53^. AUC along with sensitivity and specificity values were calculated. The average of the 10-fold CV’s performance was used to determine the performance of the prediction models.

### Metabolomics pathway topology analysis

Third trimester metabolites that were found to be significantly different (p < 0.05) between controls and tPE patients were applied to the pathway topology search tool in MetaboAnalyst v3.0^56–58^. The pathway library chosen was that for *Homo sapiens* and all compounds in the selected pathways were used when referencing the specific metabolome. Fisher’s exact test was applied for over-representation analysis and relative “betweenness centrality” was chosen for pathway topology testing. Pathways with a p-value < 0.05 were considered to be altered due to the disease.

### Integrated pathway over-representation and network analysis

The online tool, Integrated Molecular Pathway Level Analysis (IMPaLA)^59^ was employed for the integrated metabolomics and proteomic pathway over-representation analysis. Proteins were identified using UniProt Knowledgebase (http://www.uniprot.org/), while metabolites were identified via their HMDB numbers^60^. For this analysis, only third trimester metabolites and proteins with a raw *p*-value < 0.1 were considered. Pathways were identified using different biological databases including Reactome (http://www.reactome.org), KEGG (http://www.genome.jp/kegg/) or Wikipathways (http://www.wikipathways.org). The number of overlapping genes, metabolites and raw p-values were calculated for each integrated pathway. We also applied Metscape, a Cytoscape plugin, to analyze the integrated pathway of metabolites, proteins, and it’s corresponding genes for the third trimester^22^.

### Data availability

The datasets generated during and/or analysed during the current study are available from the corresponding author on reasonable request.

## Electronic supplementary material

Supplementary Information
