# Supplementary material for: Integrated Proteomic and Metabolomic prediction of Term Preeclampsia
Source: Sci Rep. 2017 Nov 23;7:16189. doi: 10.1038/s41598-017-15882-9 (PMC5700929; doi:10.1038/s41598-017-15882-9)
Supplement: Supplementary file 1 — Supplementary Information [file 41598_2017_15882_MOESM1_ESM.pdf]

Integrated Proteomic and Metabolomic prediction of Term Preeclampsia

Ray Bahado-Singh[1*], Liona C. Poon[2,3], Ali Yilmaz[1], Argyro Syngelaki[2], Onur Turkoglu[1], Praveen Kumar[1], Joseph Kirma[4], Matthew Allos[4], Veronica Accurti[2], Jiansheng Li[5], Peng Zhao[5], Stewart F. Graham[1], David R. Cool[6,7], Kypros Nicolaides[2]

(1) Department of Obstetrics and Gynecology, Beaumont Health, Royal Oak, MI, USA

(2) Harris Birthright Research Centre for Fetal Medicine, King's College Hospital, London, UK

(3) Department of Obstetrics and Gynecology, The Chinese University of Hong Kong, Hong Kong SAR

(4) Oakland University, Rochester Hills, MI, USA

(5) Henan University of Chinese Medicine, Zhengzhou, Henan, China

(6) Department of Pharmacology & Toxicology, Wright State University, Dayton, OH, USA

(7) Department of Obstetrics & Gynecology, Wright State University, Dayton, OH, USA

Suplementary Table S1. Univariate analysis of selected* DI-LC-MS/MS and NMR metabolites in first trimester

DI-LC-MS/MS Metabolite*	Mean(SD)		tPE/Control	Fold Change	p-value
	Control	tPE			
Number of cases	63	35	-	-	-
Asn	47.357 (11.728)	49.834 (15.534)	Up	-1.05	0.65799
Ala	224.821 (47.818)	237.164 (55.838)	Up	-1.05	0.25734
Asp	28.719 (10.731)	31.091 (13.908)	Up	-1.08	0.19404
Cit	17.037 (6.065)	18.903 (7.471)	Up	-1.11	0.19291
Gln	489.087 (121.095)	514.737 (135.452)	Up	-1.05	0.44625
Glu	219.197 (1125.484)	117.364 (350.486)	Down	1.87	**0.03694**
Gly	198.884 (59.851)	212.184 (61.130)	Up	-1.07	0.24519
His	91.241 (22.790)	97.168 (28.042)	Up	-1.06	0.46195
Ile	65.919 (20.427)	72.505 (34.486)	Up	-1.1	0.75468
Leu	112.488 (40.221)	126.037 (53.623)	Up	-1.12	0.31293
Lys	196.225 (57.020)	203.737 (75.692)	Up	-1.04	0.95068
Met	24.048 (6.036)	26.597 (10.195)	Up	-1.11	0.36722
Orn	37.649 (16.784)	41.242 (17.134)	Up	-1.1	0.23609
Phe	76.677 (23.492)	84.784 (33.601)	Up	-1.11	0.28423
Pro	147.122 (46.829)	158.750 (76.173)	Up	-1.08	0.64624
Ser	118.636 (27.594)	129.597 (42.484)	Up	-1.09	0.28123
Thr	118.642 (27.304)	135.118 (44.842)	Up	-1.14	0.05945
Trp	68.193 (24.237)	74.613 (33.398)	Up	-1.09	0.55576
Tyr	58.796 (18.227)	61.476 (25.140)	Up	-1.05	0.87328
Val	199.610 (57.785)	203.921 (79.350)	Up	-1.02	0.85791
Ac-Orn	0.228 (0.338)	0.409 (0.822)	Up	-1.79	0.20288
ADMA	1.533 (2.039)	1.364 (2.003)	Down	1.12	0.27410
alpha-AAA	0.603 (0.454)	0.622 (0.358)	Up	-1.03	0.40463
c4-OH-Pro	0.221 (0.339)	0.256 (0.348)	Up	-1.16	0.41128
Carnosine	0.059 (0.073)	0.061 (0.077)	Up	-1.04	0.84099
Creatinine	49.651 (14.447)	57.445 (22.357)	Up	-1.16	0.05786
DOPA	0.154 (0.102)	0.145 (0.110)	Down	1.06	0.62280
Dopamine	0.122 (0.087)	0.154 (0.186)	Up	-1.26	0.91648
Histamine	0.227 (0.136)	0.241 (0.132)	Up	-1.06	0.77884
Kynurenine	1.718 (0.726)	2.102 (1.190)	Up	-1.22	0.09954
Met-SO	0.636 (0.355)	0.619 (0.320)	Down	1.03	0.82230
Nitro-Tyr	0.045 (0.049)	0.045 (0.054)	Up	-1.01	0.86666
Putrescine	1.766 (1.411)	1.258 (2.194)	Down	1.4	**0.00150**
Sarcosine	16.750 (3.737)	18.218 (4.737)	Up	-1.09	0.07391
Serotonin	1.040 (0.597)	1.158 (0.657)	Up	-1.11	0.26423
Spermidine	0.156 (0.102)	0.164 (0.095)	Up	-1.05	0.41207
Spermine	1.876 (2.193)	1.483 (2.082)	Down	1.27	0.38613
t4-OH-Pro	8.241 (3.989)	10.650 (6.845)	Up	-1.29	0.10295
Taurine	47.019 (20.420)	45.584 (19.689)	Down	1.03	0.71301
SDMA	0.825 (0.826)	0.771 (0.812)	Down	1.07	0.98702
C0	26.649 (7.117)	26.755 (8.164)	Up	-1	0.86559
C10	0.105 (0.086)	0.097 (0.052)	Down	1.08	0.87071
C10:1	0.238 (0.062)	0.246 (0.073)	Up	-1.03	0.58896
C12	0.046 (0.020)	0.046 (0.016)	Down	1.01	0.92734
C12-DC	0.047 (0.006)	0.047 (0.005)	Down	1.01	0.88829

C12:1	0.201 (0.051)	0.212 (0.063)	Up	-1.06	0.30802
C14	0.020 (0.005)	0.020 (0.006)	Down	1.02	0.54804
C14:1	0.075 (0.023)	0.080 (0.027)	Up	-1.07	0.49211
C14:1-OH	0.007 (0.002)	0.007 (0.003)	Up	-1.06	0.61011
C14:2	0.011 (0.008)	0.011 (0.007)	Up	-1.04	0.49459
C14:2-OH	0.003 (0.001)	0.004 (0.002)	Up	-1.16	0.08131
C16	0.047 (0.017)	0.050 (0.021)	Up	-1.07	0.50865
C16-OH	0.003 (0.002)	0.004 (0.002)	Up	-1.05	0.47070
C16:1	0.011 (0.005)	0.011 (0.005)	Up	-1.06	0.32038
C16:1-OH	0.008 (0.002)	0.009 (0.003)	Up	-1.09	0.20642
C16:2	0.005 (0.002)	0.005 (0.002)	Up	-1.04	0.63880
C16:2-OH	0.012 (0.002)	0.012 (0.002)	Down	1.01	0.70476
C18	0.017 (0.006)	0.018 (0.007)	Up	-1.03	0.87813
C18:1	0.047 (0.019)	0.049 (0.022)	Up	-1.04	0.78699
C18:1-OH	0.004 (0.002)	0.004 (0.002)	Down	1.06	0.96508
C18:2	0.016 (0.007)	0.017 (0.007)	Up	-1.07	0.41513
C2	2.856 (1.129)	2.984 (1.092)	Up	-1.04	0.27125
C3	0.230 (0.086)	0.232 (0.090)	Up	-1.01	0.90414
C3-OH	0.006 (0.003)	0.006 (0.003)	Up	-1.08	0.28903
C31	0.009 (0.003)	0.009 (0.003)	Up	-1.05	0.80330
C4	0.142 (0.104)	0.132 (0.130)	Down	1.07	0.06881
C3-DC/C4-OH	0.033 (0.017)	0.033 (0.015)	Down	1	0.68626
C4:1	0.010 (0.006)	0.011 (0.005)	Up	-1.17	0.22968
C5	0.088 (0.034)	0.086 (0.046)	Down	1.03	0.31135
C5-M-DC	0.017 (0.006)	0.017 (0.005)	Up	-1	0.69223
C5-OH/C3-DC-M	0.022 (0.005)	0.022 (0.004)	Up	-1.03	0.26575
C5:1	0.012 (0.005)	0.011 (0.005)	Down	1.08	0.32978
C5:1-DC	0.006 (0.004)	0.007 (0.004)	Up	-1.13	0.08614
C6/C4:1-DC	0.026 (0.012)	0.024 (0.008)	Down	1.04	0.69328
C5-DC/C6-OH	0.010 (0.004)	0.010 (0.004)	Down	1.04	0.48946
C6:1	0.011 (0.005)	0.011 (0.004)	Up	-1.03	0.27924
C7-DC	0.011 (0.006)	0.012 (0.007)	Up	-1.07	0.76401
C8	0.069 (0.039)	0.064 (0.021)	Down	1.09	0.86810
C9	0.019 (0.010)	0.019 (0.010)	Down	1.03	0.69084
lysoPC a C14:0	3.550 (1.261)	3.611 (1.700)	Up	-1.02	0.46791
lysoPC a C16:0	111.001 (61.540)	119.018 (77.035)	Up	-1.07	0.64392
lysoPC a C16:1	1.920 (1.059)	2.250 (1.731)	Up	-1.17	0.37949
lysoPC a C17:0	2.322 (1.419)	2.328 (1.882)	Up	-1	0.39920
lysoPC a C18:0	26.088 (15.248)	27.842 (18.545)	Up	-1.07	0.94550
lysoPC a C18:1	17.900 (10.640)	18.395 (13.594)	Up	-1.03	0.67932
lysoPC a C18:2	20.818 (13.003)	22.541 (18.533)	Up	-1.08	0.84516
lysoPC a C20:3	1.407 (0.858)	1.505 (1.051)	Up	-1.07	0.68649
lysoPC a C20:4	4.278 (2.317)	4.645 (3.466)	Up	-1.09	0.69369
lysoPC a C24:0	0.149 (0.060)	0.151 (0.047)	Up	-1.01	0.55355
lysoPC a C26:0	0.187 (0.078)	0.191 (0.079)	Up	-1.02	0.88869
lysoPC a C26:1	0.078 (0.031)	0.076 (0.022)	Down	1.03	0.92218
lysoPC a C28:0	0.197 (0.080)	0.190 (0.075)	Down	1.04	0.50876
lysoPC a C28:1	0.256 (0.117)	0.233 (0.095)	Down	1.1	0.25323
PC aa C24:0	0.075 (0.026)	0.083 (0.033)	Up	-1.1	0.27545
PC aa C26:0	0.636 (0.118)	0.656 (0.146)	Up	-1.03	0.72518
PC aa C28:1	2.246 (0.839)	2.183 (0.856)	Down	1.03	0.56892

PC aa C30:0	3.724 (1.886)	3.737 (2.119)	Up	-1	0.71544
PC aa C32:0	15.032 (4.931)	15.790 (6.029)	Up	-1.05	0.53411
PC aa C32:1	12.337 (6.966)	12.425 (7.617)	Up	-1.01	0.90930
PC aa C32:2	4.062 (2.539)	3.793 (2.160)	Down	1.07	0.78954
PC aa C32:3	0.369 (0.143)	0.388 (0.200)	Up	-1.05	0.66745
PC aa C34:1	230.780 (90.540)	238.697 (96.836)	Up	-1.03	0.61159
PC aa C34:2	407.797 (105.758)	419.526 (93.423)	Up	-1.03	0.71301
PC aa C34:3	11.032 (4.877)	11.156 (5.576)	Up	-1.01	0.92738
PC aa C34:4	1.277 (0.633)	1.314 (0.767)	Up	-1.03	0.98182
PC aa C36:0	4.597 (1.950)	5.027 (2.056)	Up	-1.09	0.34354
PC aa C36:1	39.072 (16.483)	40.442 (16.945)	Up	-1.04	0.56673
PC aa C36:2	214.390 (81.805)	219.632 (82.465)	Up	-1.02	0.84260
PC aa C36:3	120.383 (45.238)	124.055 (50.292)	Up	-1.03	0.98182
PC aa C36:4	186.399 (63.553)	198.853 (88.399)	Up	-1.07	0.64859
PC aa C36:5	16.541 (11.881)	16.464 (10.913)	Down	1	0.96365
PC aa C36:6	0.698 (0.451)	0.719 (0.376)	Up	-1.03	0.48405
PC aa C38:0	3.644 (1.513)	3.996 (1.523)	Up	-1.1	0.22471
PC aa C38:3	41.346 (14.946)	45.811 (16.923)	Up	-1.11	0.17468
PC aa C38:4	90.486 (30.555)	100.434 (43.763)	Up	-1.11	0.35021
PC aa C38:5	38.178 (14.802)	41.424 (18.654)	Up	-1.09	0.30215
PC aa C38:6	103.094 (43.397)	112.882 (37.806)	Up	-1.09	0.09240
PC aa C40:1	0.376 (0.121)	0.402 (0.113)	Up	-1.07	0.20313
PC aa C40:2	0.333 (0.134)	0.360 (0.146)	Up	-1.08	0.42707
PC aa C40:3	0.556 (0.183)	0.613 (0.235)	Up	-1.1	0.38126
PC aa C40:4	3.899 (1.516)	4.356 (1.749)	Up	-1.12	0.11073
PC aa C40:5	7.850 (2.998)	8.996 (3.823)	Up	-1.15	0.06217
PC aa C40:6	31.612 (12.589)	36.837 (13.606)	Up	-1.17	**0.04324**
PC aa C42:0	0.860 (0.383)	0.884 (0.354)	Up	-1.03	0.71059
PC aa C42:1	0.362 (0.143)	0.380 (0.138)	Up	-1.05	0.52137
PC aa C42:2	0.258 (0.090)	0.274 (0.114)	Up	-1.06	0.53627
PC aa C42:4	0.230 (0.074)	0.249 (0.077)	Up	-1.08	0.22922
PC aa C42:5	0.456 (0.169)	0.518 (0.191)	Up	-1.14	0.10022
PC aa C42:6	0.585 (0.211)	0.638 (0.227)	Up	-1.09	0.20083
PC ae C30:0	0.369 (0.158)	0.352 (0.148)	Down	1.05	0.49632
PC ae C30:2	0.063 (0.020)	0.067 (0.023)	Up	-1.07	0.45390
PC ae C32:1	2.496 (0.886)	2.576 (0.849)	Up	-1.03	0.59798
PC ae C32:2	0.648 (0.207)	0.672 (0.239)	Up	-1.04	0.85026
PC ae C34:0	1.614 (0.665)	1.604 (0.767)	Down	1.01	0.69849
PC ae C34:1	10.768 (4.252)	10.583 (4.349)	Down	1.02	0.78703
PC ae C34:2	12.974 (4.983)	12.315 (4.575)	Down	1.05	0.45997
PC ae C34:3	7.907 (2.931)	8.187 (2.878)	Up	-1.04	0.60025
PC ae C36:0	0.673 (0.230)	0.716 (0.233)	Up	-1.06	0.33532
PC ae C36:1	22.396 (8.818)	21.964 (9.895)	Down	1.02	0.66981
PC ae C36:2	16.272 (6.406)	15.959 (7.349)	Down	1.02	0.52348
PC ae C36:3	7.002 (2.630)	7.044 (2.760)	Up	-1.01	0.83244
PC ae C36:4	15.654 (5.467)	15.972 (6.614)	Up	-1.02	0.89640
PC ae C36:5	9.210 (3.272)	9.975 (4.364)	Up	-1.08	0.63462
PC ae C38:0	1.656 (0.786)	1.773 (0.737)	Up	-1.07	0.30828
PC ae C38:2	0.994 (0.696)	0.927 (0.598)	Down	1.07	0.63221
PC ae C38:3	7.388 (2.761)	7.480 (3.338)	Up	-1.01	0.71059
PC ae C38:4	11.075 (3.915)	11.421 (5.537)	Up	-1.03	0.67218

PC ae C38:5	13.371 (4.460)	13.940 (5.842)	Up	-1.04	0.97403
PC ae C38:6	5.884 (2.356)	6.297 (2.472)	Up	-1.07	0.44627
PC ae C40:1	1.444 (0.586)	1.543 (0.598)	Up	-1.07	0.44237
PC ae C40:2	2.631 (1.073)	2.655 (1.161)	Up	-1.01	0.98182
PC ae C40:3	1.899 (0.661)	1.908 (0.695)	Up	-1	0.95069
PC ae C40:4	2.218 (0.768)	2.267 (0.918)	Up	-1.02	0.79204
PC ae C40:5	4.356 (1.582)	4.517 (1.930)	Up	-1.04	0.94291
PC ae C40:6	5.066 (2.117)	5.292 (2.179)	Up	-1.04	0.63463
PC ae C42:0	1.233 (0.294)	1.280 (0.298)	Up	-1.04	0.58220
PC ae C42:1	0.467 (0.149)	0.506 (0.133)	Up	-1.08	0.17263
PC ae C42:2	0.586 (0.244)	0.616 (0.248)	Up	-1.05	0.60024
PC ae C42:3	1.077 (0.426)	1.065 (0.354)	Down	1.01	0.87328
PC ae C42:4	0.961 (0.404)	0.926 (0.391)	Down	1.04	0.45801
PC ae C42:5	2.517 (0.882)	2.501 (1.039)	Down	1.01	0.48607
PC ae C44:3	0.149 (0.054)	0.150 (0.046)	Up	-1	0.74232
PC ae C44:4	0.423 (0.171)	0.403 (0.162)	Down	1.05	0.42330
PC ae C44:5	1.908 (0.828)	1.813 (0.886)	Down	1.05	0.33042
PC ae C44:6	1.213 (0.496)	1.190 (0.467)	Down	1.02	0.70815
SM OH C14:1	6.803 (2.349)	6.833 (2.782)	Up	-1	0.86560
SM OH C16:1	3.796 (1.295)	4.039 (1.783)	Up	-1.06	0.77454
SM OH C22:1	14.057 (4.779)	14.502 (5.276)	Up	-1.03	0.80964
SM OH C22:2	12.990 (4.270)	13.748 (4.994)	Up	-1.06	0.62076
SM OH C24:1	1.411 (0.480)	1.477 (0.556)	Up	-1.05	0.71058
SM C16:0	122.513 (38.003)	133.550 (45.573)	Up	-1.09	0.27694
SM C16:1	17.865 (5.447)	19.212 (6.476)	Up	-1.08	0.35357
SM C18:0	21.781 (7.177)	25.471 (11.474)	Up	-1.17	0.17988
SM C18:1	12.101 (3.854)	14.465 (6.649)	Up	-1.2	0.14566
SM C20:2	0.651 (0.222)	0.729 (0.361)	Up	-1.12	0.34189
SM C24:0	19.609 (6.577)	20.868 (6.754)	Up	-1.06	0.37420
SM C24:1	68.625 (22.179)	76.308 (29.204)	Up	-1.11	0.29012
SM C26:0	0.177 (0.064)	0.183 (0.074)	Up	-1.03	0.84770
SM C26:1	0.460 (0.173)	0.493 (0.202)	Up	-1.07	0.55576
H1	4186.333 (969.611)	4690.447 (1532.568)	Up	-1.12	0.07608
**NMR Metabolite***	**Mean(SD)**		**Control/tPE**	**Fold Change**	**p-value**
1-Methylhistidine	145.900 (42.689)	138.574 (36.956)	Down	1.05	0.37563 (t)
2-Hydroxybutyric acid	15.754 (7.747)	15.763 (6.277)	Up	-1	0.90414
Acetic acid	13.907 (6.139)	12.184 (5.235)	Down	1.14	0.14657
Betaine	21.629 (9.087)	22.461 (6.569)	Up	-1.04	0.37071
Acetoacetate	15.126 (18.591)	11.071 (5.660)	Down	1.37	0.59119
Carnitine	17.764 (5.559)	16.439 (4.168)	Down	1.08	0.40833
Creatine	29.003 (15.198)	29.118 (13.165)	Up	-1	0.68889
Choline	2.803 (1.380)	2.929 (1.419)	Up	-1.04	0.65533 (t)
Ethanol	14.796 (14.289)	11.368 (4.748)	Down	1.3	0.90671
D-Glucose	3600.652 (1049.317)	3733.824 (1154.655)	Up	-1.04	0.74235
Glycerol	268.928 (79.839)	283.321 (87.377)	Up	-1.05	0.56674
Formate	28.367 (6.631)	28.108 (6.396)	Down	1.01	0.96106
Hypoxanthine	0.257 (0.112)	0.261 (0.108)	Up	-1.02	0.67726
L-Lactic acid	1834.859 (946.152)	1854.847 (1170.146)	Up	-1.01	0.74235
Pyruvic acid	72.352 (25.919)	82.576 (37.067)	Up	-1.14	0.30217
Succinate	22.423 (5.285)	20.892 (5.370)	Down	1.07	0.15684 (t)
Urea	140.075 (51.625)	109.618 (31.091)	Down	1.28	**0.00201**

3-Hydroxybutyric acid	29.978 (55.599)	19.339 (11.040)	Down	1.55	0.97663
L-Arginine	41.296 (18.170)	41.984 (15.621)	Up	-1.02	0.84432 (t)
Creatinine.1	39.020 (9.827)	41.979 (13.448)	Up	-1.08	0.43660
Malonate	6.284 (2.629)	5.350 (2.059)	Down	1.17	0.07872
Isopropyl alcohol	4.620 (13.698)	2.966 (4.003)	Down	1.56	0.27350
Acetone	9.971 (4.759)	8.774 (3.442)	Down	1.14	0.27405
Methanol	1777.174 (430.644)	1846.342 (557.485)	Up	-1.04	0.54707
Propylene glycol	0.616 (0.262)	0.592 (0.248)	Down	1.04	0.66591
Dimethyl sulfone	5.993 (2.895)	4.774 (2.109)	Down	1.26	**0.03720**
Isobutyric acid	6.232 (2.540)	5.497 (1.901)	Down	1.13	0.23336

p-value is calculated with Wilcoxon Mann Whitney test as a default.
p-value with (t) is calculated by the t-test

Supplementary Table S2. Univariate analysis of selected* DI-LC-MS/MS and NMR metabolites in third trimester

DI-LC-MS/MS Metabolite*	Mean (SD)		tPE / Control	Fold Change	p-value
	Control	tPE			
Number of cases	63	35	-	-	-
Ala	412.368 (112.859)	408.435 (100.695)	Down	-1.01	0.9662
Asn	55.018 (16.516)	56.009 (13.566)	Up	1.02	0.4214
Asp	48.450 (16.643)	43.574 (14.788)	Down	-1.11	0.1448
Cit	19.522 (7.862)	16.348 (4.914)	Down	-1.19	0.0532
Gln	510.684 (140.901)	500.275 (97.903)	Down	-1.02	0.9041
Glu	94.534 (36.977)	84.184 (30.972)	Down	-1.12	0.1896
Gly	238.368 (70.526)	223.681 (53.496)	Down	-1.07	0.5427
His	109.445 (37.361)	105.278 (23.377)	Down	-1.04	0.8274
Ile	66.711 (32.989)	61.722 (19.072)	Down	-1.08	0.8938
Leu	115.358 (52.096)	111.107 (35.601)	Down	-1.04	0.6323
Lys	210.737 (69.837)	210.342 (55.629)	Down	-1	0.7033
Met	27.324 (11.213)	25.151 (6.080)	Down	-1.09	0.4840
Orn	50.055 (41.693)	46.670 (19.552)	Down	-1.07	0.6674
Phe	94.024 (30.341)	87.932 (23.575)	Down	-1.07	0.6533
Pro	162.658 (47.073)	163.941 (43.588)	Up	1.01	0.6369
Ser	158.695 (53.404)	149.929 (41.017)	Down	-1.06	0.6937
Thr	184.576 (56.082)	183.978 (48.045)	Down	-1	0.9538 (t)
Trp	58.795 (22.776)	57.581 (16.921)	Down	-1.02	0.6674
Tyr	60.550 (23.812)	57.545 (16.211)	Down	-1.05	0.9818
Val	183.058 (69.107)	180.493 (45.777)	Down	-1.01	0.2642
Ac-Orn	0.224 (0.418)	0.215 (0.371)	Down	-1.04	0.4706
ADMA	3.537 (14.673)	3.085 (11.662)	Down	-1.15	0.5667
alpha-AAA	0.625 (0.382)	0.518 (0.390)	Down	-1.21	0.2053
c4-OH-Pro	0.170 (0.278)	0.219 (0.328)	Up	1.29	0.7919
Carnosine	0.047 (0.065)	0.060 (0.075)	Up	1.29	0.6921
Creatinine	48.721 (14.836)	46.543 (11.758)	Down	-1.05	0.8503
DOPA	0.125 (0.105)	0.170 (0.106)	Up	1.36	0.0357
Dopamine	0.155 (0.190)	0.155 (0.206)	Up	1	0.6370
Histamine	0.208 (0.124)	0.226 (0.134)	Up	1.09	0.4209
Kynurenine	1.888 (0.665)	1.783 (0.506)	Down	-1.06	0.5384
Met-SO	0.752 (0.364)	0.711 (0.342)	Down	-1.06	0.6416
Nitro-Tyr	0.042 (0.048)	0.048 (0.052)	Up	1.12	0.7383
Putrescine	17.629 (35.149)	47.222 (120.514)	Up	2.68	0.2322
Sarcosine	19.874 (5.446)	19.338 (4.858)	Down	-1.03	0.7596
Serotonin	1.113 (0.589)	0.837 (0.483)	Down	-1.33	**0.0205**
Spermidine	0.145 (0.173)	0.122 (0.078)	Down	-1.19	0.8554
Spermine	1.984 (2.611)	1.784 (2.262)	Down	-1.11	0.8415
t4-OH-Pro	14.560 (11.681)	11.082 (7.872)	Down	-1.31	**0.0139**
Taurine	74.984 (27.859)	72.928 (36.742)	Down	-1.03	0.7644 (t)
SDMA	1.995 (7.595)	1.631 (5.784)	Down	-1.22	0.8477
C0	16.350 (4.634)	16.436 (4.122)	Up	1.01	0.9714
C10	0.099 (0.057)	0.101 (0.052)	Up	1.02	0.4840
C10:1	0.224 (0.064)	0.245 (0.072)	Up	1.1	0.2161
C10:2	0.034 (0.013)	0.034 (0.014)	Up	1.01	0.8170
C12	0.047 (0.019)	0.045 (0.014)	Down	-1.03	0.9662
C12-DC	0.046 (0.006)	0.046 (0.009)	Down	-1.02	0.1207
C12:1	0.182 (0.052)	0.199 (0.054)	Up	1.09	0.1457

C14	0.023 (0.010)	0.021 (0.006)	Down	-1.07	0.6336
C14:1	0.093 (0.031)	0.095 (0.025)	Up	1.02	0.3937
C14:1-OH	0.007 (0.003)	0.006 (0.002)	Down	-1.07	0.9187
C14:2	0.012 (0.010)	0.011 (0.007)	Down	-1.12	0.5699
C14:2-OH	0.004 (0.002)	0.004 (0.002)	Down	-1.15	0.3725
C16	0.054 (0.027)	0.049 (0.019)	Down	-1.1	0.7324
C16-OH	0.004 (0.002)	0.004 (0.002)	Down	-1.07	0.9492
C16:1	0.012 (0.008)	0.011 (0.004)	Down	-1.18	0.2953
C16:1-OH	0.008 (0.003)	0.009 (0.003)	Up	1.08	0.6476
C16:2	0.006 (0.003)	0.005 (0.002)	Down	-1.04	0.8331
C16:2-OH	0.012 (0.002)	0.012 (0.002)	Up	1.03	0.2359
C18	0.019 (0.012)	0.017 (0.008)	Down	-1.11	0.7394
C18:1	0.058 (0.031)	0.051 (0.020)	Down	-1.14	0.4500
C18:1-OH	0.005 (0.003)	0.004 (0.002)	Down	-1.14	0.6649
C18:2	0.020 (0.010)	0.017 (0.008)	Down	-1.17	0.2541
C2	2.348 (0.905)	2.119 (0.626)	Down	-1.11	0.1702 (t)
C3	0.143 (0.065)	0.147 (0.053)	Up	1.03	0.3638
C3-OH	0.006 (0.003)	0.006 (0.003)	Up	1.04	0.9659
C31	0.009 (0.004)	0.008 (0.004)	Down	-1.14	0.1812
C4	0.086 (0.042)	0.089 (0.032)	Up	1.03	0.2684
C3-DC/C4-OH	0.042 (0.017)	0.041 (0.017)	Down	-1.04	0.6508
C4:1	0.011 (0.006)	0.011 (0.006)	Up	1.01	0.6378
C5	0.066 (0.029)	0.066 (0.026)	Down	-1.01	0.6556
C5-M-DC	0.017 (0.005)	0.017 (0.005)	Up	1.02	0.4663
C5-OH/C3-DC-M	0.021 (0.005)	0.020 (0.004)	Down	-1.05	0.2900
C5:1	0.013 (0.004)	0.013 (0.007)	Down	-1	0.4963
C5:1-DC	0.006 (0.003)	0.006 (0.004)	Down	-1.11	0.3559
C6/C4:1-DC	0.024 (0.008)	0.025 (0.008)	Up	1.03	0.7494
C5-DC/C6-OH	0.011 (0.004)	0.011 (0.004)	Up	1	0.8190
C6:1	0.010 (0.004)	0.010 (0.004)	Down	-1.01	0.8413
C7-DC	0.011 (0.005)	0.011 (0.004)	Down	-1.02	0.9922
C8	0.065 (0.024)	0.065 (0.019)	Down	-1.01	0.7032
C9	0.019 (0.007)	0.021 (0.011)	Up	1.12	0.5357
lysoPC a C14:0	3.231 (0.941)	3.481 (0.956)	Up	1.08	0.1036
lysoPC a C16:0	105.537 (44.916)	105.030 (51.848)	Down	-1	0.9792
lysoPC a C16:1	1.737 (0.912)	1.827 (0.930)	Up	1.05	0.4560
lysoPC a C17:0	1.526 (0.876)	1.614 (0.801)	Up	1.06	0.2670
lysoPC a C18:0	19.460 (8.048)	19.177 (9.491)	Down	-1.01	0.8122
lysoPC a C18:1	12.127 (4.867)	12.283 (6.052)	Up	1.01	0.9507
lysoPC a C18:2	10.412 (3.778)	10.823 (4.956)	Up	1.04	0.8682
lysoPC a C20:3	0.995 (0.471)	0.998 (0.468)	Up	1	0.9196
lysoPC a C20:4	2.246 (0.871)	2.518 (1.110)	Up	1.12	0.2101
lysoPC a C24:0	0.115 (0.046)	0.121 (0.043)	Up	1.05	0.3502
lysoPC a C26:0	0.212 (0.129)	0.191 (0.076)	Down	-1.11	0.8990
lysoPC a C26:1	0.065 (0.024)	0.068 (0.024)	Up	1.04	0.6154 (t)
lysoPC a C28:0	0.176 (0.072)	0.182 (0.062)	Up	1.03	0.4881
lysoPC a C28:1	0.241 (0.097)	0.257 (0.097)	Up	1.07	0.3021
PC aa C24:0	0.079 (0.039)	0.074 (0.040)	Down	-1.06	0.3401
PC aa C26:0	0.762 (0.208)	0.703 (0.139)	Down	-1.08	0.2439
PC aa C28:1	2.820 (1.014)	3.062 (1.251)	Up	1.09	0.4620
PC aa C30:0	5.092 (2.352)	5.370 (2.724)	Up	1.05	0.7154
PC aa C32:0	20.805 (7.688)	19.944 (6.821)	Down	-1.04	0.8452
PC aa C32:1	24.678 (18.382)	23.238 (15.644)	Down	-1.06	0.8861

PC aa C32:2	6.608 (3.953)	6.647 (3.965)	Up	1.01	0.9559
PC aa C32:3	0.504 (0.189)	0.522 (0.210)	Up	1.03	0.5980
PC aa C34:1	351.053 (122.771)	347.159 (102.069)	Down	-1.01	0.8610 (t)
PC aa C34:2	482.421 (97.003)	497.159 (94.373)	Up	1.03	0.3502
PC aa C34:3	16.590 (7.660)	16.983 (7.333)	Up	1.02	0.5645
PC aa C34:4	1.529 (0.714)	1.676 (0.793)	Up	1.1	0.2857
PC aa C36:0	5.351 (1.704)	4.874 (1.664)	Down	-1.1	0.0739
PC aa C36:1	50.421 (19.590)	48.867 (17.334)	Down	-1.03	0.8861
PC aa C36:2	264.526 (87.847)	252.420 (80.453)	Down	-1.05	0.5129
PC aa C36:3	174.489 (80.235)	167.335 (60.037)	Down	-1.04	0.8810
PC aa C36:4	227.726 (86.604)	238.339 (80.515)	Up	1.05	0.2297
PC aa C36:5	16.841 (10.473)	16.348 (8.566)	Down	-1.03	0.9377
PC aa C36:6	0.804 (0.442)	0.825 (0.460)	Up	1.03	0.8656
PC aa C38:0	4.479 (1.224)	4.282 (1.531)	Down	-1.05	0.2297
PC aa C38:3	52.766 (22.030)	48.962 (16.532)	Down	-1.08	0.6793
PC aa C38:4	89.713 (33.181)	90.696 (30.527)	Up	1.01	0.5867
PC aa C38:5	41.184 (17.086)	39.777 (12.803)	Down	-1.04	0.7596
PC aa C38:6	131.350 (52.936)	126.239 (48.916)	Down	-1.04	0.7276
PC aa C40:1	0.440 (0.122)	0.424 (0.136)	Down	-1.04	0.3098
PC aa C40:2	0.414 (0.155)	0.402 (0.135)	Down	-1.03	0.7057
PC aa C40:3	0.697 (0.260)	0.647 (0.207)	Down	-1.08	0.5046
PC aa C40:4	4.886 (2.202)	4.592 (1.525)	Down	-1.06	0.9766
PC aa C40:5	9.732 (4.207)	8.937 (3.086)	Down	-1.09	0.6231
PC aa C40:6	36.100 (15.154)	33.152 (12.560)	Down	-1.09	0.3795
PC aa C42:0	0.967 (0.283)	0.998 (0.409)	Up	1.03	0.9481
PC aa C42:1	0.415 (0.135)	0.410 (0.169)	Down	-1.01	0.5667
PC aa C42:2	0.295 (0.097)	0.277 (0.085)	Down	-1.06	0.3067
PC aa C42:4	0.307 (0.104)	0.296 (0.093)	Down	-1.03	0.6323
PC aa C42:5	0.685 (0.275)	0.598 (0.204)	Down	-1.14	0.1269
PC aa C42:6	0.692 (0.255)	0.658 (0.225)	Down	-1.05	0.6254
PC ae C30:0	0.394 (0.164)	0.442 (0.193)	Up	1.12	0.2601
PC ae C30:2	0.076 (0.028)	0.079 (0.026)	Up	1.04	0.4346
PC ae C32:1	2.982 (1.058)	2.868 (0.932)	Down	-1.04	0.9481
PC ae C32:2	0.755 (0.243)	0.709 (0.205)	Down	-1.06	0.4385
PC ae C34:0	1.797 (0.699)	1.901 (0.730)	Up	1.06	0.5623
PC ae C34:1	13.449 (5.306)	14.006 (4.744)	Up	1.04	0.2991
PC ae C34:2	15.377 (5.308)	16.022 (5.477)	Up	1.04	0.7276
PC ae C34:3	8.337 (2.943)	7.963 (2.882)	Down	-1.05	0.6961
PC ae C36:0	0.773 (0.226)	0.721 (0.215)	Down	-1.07	0.3083
PC ae C36:1	25.922 (10.031)	27.361 (9.440)	Up	1.06	0.3974
PC ae C36:2	18.276 (6.963)	19.214 (7.322)	Up	1.05	0.6580
PC ae C36:3	8.318 (2.901)	8.357 (2.772)	Up	1	0.9740
PC ae C36:4	16.414 (4.811)	17.120 (5.459)	Up	1.04	0.7399
PC ae C36:5	9.735 (3.169)	9.504 (3.409)	Down	-1.02	0.6323
PC ae C38:0	1.767 (0.776)	1.740 (0.697)	Down	-1.02	0.9714
PC ae C38:2	1.113 (0.837)	0.970 (0.851)	Down	-1.15	0.2412
PC ae C38:3	8.446 (3.678)	8.624 (3.042)	Up	1.02	0.4780
PC ae C38:4	10.949 (3.781)	12.146 (4.155)	Up	1.11	0.1636
PC ae C38:5	14.334 (4.076)	14.727 (4.428)	Up	1.03	0.7057
PC ae C38:6	6.559 (1.910)	6.362 (2.143)	Down	-1.03	0.3884
PC ae C40:1	1.690 (0.681)	1.554 (0.563)	Down	-1.09	0.3052
PC ae C40:2	2.891 (1.119)	3.085 (1.164)	Up	1.07	0.3795
PC ae C40:3	2.125 (0.844)	2.247 (0.771)	Up	1.06	0.2670

PC ae C40:4	2.325 (0.807)	2.556 (0.866)	Up	1.1	0.1606
PC ae C40:5	4.613 (1.452)	4.880 (1.525)	Up	1.06	0.3707
PC ae C40:6	5.133 (1.750)	5.338 (1.939)	Up	1.04	0.6937
PC ae C42:0	1.363 (0.369)	1.352 (0.311)	Down	-1.01	0.7130
PC ae C42:1	0.552 (0.195)	0.525 (0.164)	Down	-1.05	0.6486
PC ae C42:2	0.694 (0.255)	0.677 (0.239)	Down	-1.03	0.7374
PC ae C42:3	1.428 (0.533)	1.425 (0.553)	Down	-1	0.9145
PC ae C42:4	1.041 (0.432)	1.138 (0.459)	Up	1.09	0.2235
PC ae C42:5	2.567 (0.809)	2.863 (1.009)	Up	1.12	0.0930
PC ae C44:3	0.169 (0.063)	0.171 (0.060)	Up	1.01	0.7130
PC ae C44:4	0.461 (0.186)	0.494 (0.208)	Up	1.07	0.4366
PC ae C44:5	1.866 (0.693)	2.142 (0.908)	Up	1.15	0.0949
PC ae C44:6	1.239 (0.368)	1.331 (0.542)	Up	1.07	0.5980
SM OH C14:1	8.147 (3.149)	8.986 (3.301)	Up	1.1	0.2101
SM OH C16:1	4.640 (1.772)	4.765 (1.679)	Up	1.03	0.6557
SM OH C22:1	17.729 (5.850)	18.593 (5.339)	Up	1.05	0.3777
SM OH C22:2	15.176 (4.939)	16.216 (4.563)	Up	1.07	0.2090
SM OH C24:1	1.466 (0.462)	1.511 (0.464)	Up	1.03	0.5514
SM C16:0	163.442 (49.334)	160.891 (44.475)	Down	-1.02	0.7081
SM C16:1	23.784 (7.239)	24.271 (7.158)	Up	1.02	0.4963
SM C18:0	31.347 (10.652)	29.800 (9.941)	Down	-1.05	0.5800
SM C18:1	16.509 (5.924)	15.743 (5.507)	Down	-1.05	0.6698
SM C20:2	0.870 (0.333)	0.885 (0.300)	Up	1.02	0.5492
SM C24:0	24.897 (7.992)	25.691 (7.677)	Up	1.03	0.4385
SM C24:1	90.171 (28.839)	89.717 (25.825)	Down	-1.01	0.5320
SM C26:0	0.177 (0.053)	0.180 (0.053)	Up	1.02	0.8758
SM C26:1	0.481 (0.145)	0.482 (0.155)	Up	1	0.9948
H1	4816.342 (1700.765)	4161.145 (1039.549)	Down	-1.16	**0.0475**
**NMR Metabolite***	**Mean (SD)**		**tPE / Control**	**Fold Change**	**p-value**
1-Methylhistidine	157.482 (43.163)	161.596 (37.417)	Up	1.03	0.3956
2-Hydroxybutyric acid	15.526 (8.217)	14.775 (6.320)	Down	-1.05	0.8810
Acetic acid	9.921 (4.869)	11.123 (3.959)	Up	1.12	**0.0280**
Betaine	19.037 (6.524)	19.975 (8.053)	Up	1.05	0.5889
Acetoacetate	19.797 (14.598)	19.774 (16.694)	Down	-1	0.8579
Carnitine	11.884 (4.572)	12.090 (4.659)	Up	1.02	0.7870
Creatine	30.039 (17.299)	30.677 (13.839)	Up	1.02	0.5514
Choline	4.526 (2.510)	4.441 (2.091)	Down	-1.02	0.8505 (t)
Ethanol	15.892 (13.441)	15.313 (8.974)	Down	-1.04	0.7423
D-Glucose	3901.161 (948.331)	3607.591 (1038.835)	Down	-1.08	0.0963
Glycerol	285.837 (109.470)	286.161 (86.358)	Up	1	0.6162
Formate	32.471 (7.331)	33.996 (8.845)	Up	1.05	0.4028
Hypoxanthine	0.176 (0.108)	0.157 (0.099)	Down	-1.13	0.2378
L-Lactic acid	2462.353 (755.787)	2698.836 (1248.030)	Up	1.1	0.7572
Pyruvic acid	94.274 (32.820)	83.465 (34.761)	Down	-1.13	0.0750
Succinate	22.395 (6.653)	22.978 (4.802)	Up	1.03	0.6354 (t)
Urea	126.721 (54.590)	121.614 (41.559)	Down	-1.04	0.9093
3-Hydroxybutyric acid	45.084 (49.066)	41.968 (50.433)	Down	-1.07	0.4328
L-Arginine	47.832 (17.044)	47.836 (14.798)	Up	1	0.9988 (t)
Creatinine .1	39.347 (9.026)	39.551 (8.440)	Up	1.01	0.9076 (t)
Malonate	6.200 (2.473)	6.003 (2.077)	Down	-1.03	0.6618 (t)
Isopropyl alcohol	4.126 (4.605)	4.790 (8.903)	Up	1.16	0.8121
Acetone	10.789 (4.225)	11.567 (5.384)	Up	1.07	0.6556

Methanol	1784.276 (333.168)	1773.242 (420.431)	Down	-1.01	0.7473
Propylene glycol	0.550 (0.244)	0.642 (0.273)	Up	1.17	0.2569
Dimethyl sulfone	4.005 (2.250)	4.941 (2.301)	Up	1.23	**0.0210**
Isobutyric acid	5.618 (2.200)	5.586 (1.823)	Down	-1.01	0.9067

p-value is calculated with Wilcoxon Mann Whitney test as a default.
p-value with (t) is calculated by the t-test

Supplementary Table S3. Univariate analysis of selected* peptides in *first trimester*

Protein	p.value	FDR	tPE vs Controls
594 m/z	**0,012**	0,996	Up
650 m/z	**0,033**	0,996	Up
636 m/z	**0,036**	0,996	Up
**TNFalpha**	0,056	0,996	Up
**SG11B**	0,057	0,996	Up
638 m/z	0,059	0,996	Up
580 m/z	0,065	0,996	Up
622 m/z	0,067	0,996	Up
1129 m/z	0,067	0,996	Up
890 m/z	0,068	0,996	Up
596 m/z	0,068	0,996	Up
1143 m/z	0,088	0,996	Up
566 m/z	0,101	0,996	Up
551 m/z	0,105	0,996	Up
506 m/z	0,107	0,996	Up
546 m/z	0,107	0,996	Up
**W5ULX4**	0,109	0,996	Up
672 m/z	0,119	0,996	Up
657 m/z	0,127	0,996	Up
603 m/z	0,132	0,996	Up
652 m/z	0,135	0,996	Up
508 m/z	0,140	0,996	Up
666 m/z	0,141	0,996	Up
547 m/z	0,143	0,996	Up
926 m/z	0,177	0,996	Up
604 m/z	0,180	0,996	Up
552 m/z	0,185	0,996	Up
667 m/z	0,187	0,996	Up
741 m/z	0,237	0,996	Up
1574 m/z	0,240	0,996	Up
740 m/z	0,242	0,996	Up
1519 m/z	0,248	0,996	Up
545 m/z	0,255	0,996	Up
632 m/z	0,265	0,996	Up
733 m/z	0,267	0,996	Up
550 m/z	0,269	0,996	Up
526 m/z	0,270	0,996	Up
905 m/z	0,279	0,996	Up
544 m/z	0,281	0,996	Up
1216 m/z	0,294	0,996	Up
588 m/z	0,295	0,996	Up

* p-value<0.3

Supplementary Table S4. Univariate analysis of selected* peptides in *third trimester*

**Proteins**	**p-value**	**FDR**	**tPE vs Controls**
F86JP	**6.6E-24**	1.2E-21	Up
STMN3	**8.7E-20**	7.8E-18	Up
HERC3	**3.6E-19**	2.2E-17	Up
TEX15	**2.0E-18**	7.5E-17	Up
CHD1L	**2.1E-18**	7.5E-17	Up
TEN1L	**2.8E-17**	8.5E-16	Up
CCL2	**5.1E-17**	1.3E-15	Up
Q5GML2	**7.4E-16**	1.7E-14	Up
DAD1	**6.0E-15**	1.2E-13	Up
WFD13	**7.3E-15**	1.3E-13	Up
1766	**1.2E-13**	1.9E-12	Up
MCHL1	**2.0E-13**	2.9E-12	Up
1639	**4.5E-12**	6.3E-11	Up
GTPB3	**6.2E-11**	8.0E-10	Up
1499 m/z	**3.8E-10**	4.6E-09	Up
SMCR5	**1.6E-09**	1.8E-08	Up
2726	**2.2E-08**	2.3E-07	Up
HLA-DR β-1	**7.0E-06**	7.0E-05	Up
1574	**4.8E-05**	4.6E-04	Down
3028	**1.3E-04**	1.1E-03	Down
1570	**3.3E-04**	2.9E-03	Down
587	**5.5E-04**	4.5E-03	Up
4282	**1.0E-03**	8.0E-03	Down
2378	**1.1E-03**	8.0E-03	Up
4267	**2.9E-03**	2.1E-02	Up
1488	**4.0E-03**	2.8E-02	Down
1314	**4.5E-03**	3.0E-02	Up
1042	**5.2E-03**	3.4E-02	Down
1945	**7.5E-03**	4.7E-02	Up
4266	**1.0E-02**	6.3E-02	Up
543	**2.1E-02**	1.2E-01	Up
741	**2.4E-02**	1.4E-01	Up
4209	**2.8E-02**	1.5E-01	Down
1626	**2.8E-02**	1.5E-01	Up
1261	**3.3E-02**	1.7E-01	Up
584	**3.6E-02**	1.8E-01	Up
1462	**4.1E-02**	2.0E-01	Up
586	6.2E-02	2.9E-01	Up
1228	6.9E-02	3.2E-01	Up
520	7.2E-02	3.2E-01	Up
585	7.5E-02	3.3E-01	Up
861	8.5E-02	3.6E-01	Down

616	9.3E-02	3.9E-01	Up
568	9.9E-02	4.0E-01	Up
709	1.0E-01	4.1E-01	Up
1263	1.1E-01	4.2E-01	Up
1434	1.1E-01	4.3E-01	Up
622	1.2E-01	4.5E-01	Up
551	1.2E-01	4.5E-01	Up
4474	1.4E-01	5.0E-01	Up
591	1.4E-01	5.0E-01	Up
1336	1.5E-01	5.2E-01	Up
544	1.6E-01	5.5E-01	Up
583	1.7E-01	5.7E-01	Up
511	1.8E-01	5.7E-01	Up
2272	1.8E-01	5.7E-01	Up
905	1.9E-01	5.8E-01	Up
1481	1.9E-01	5.8E-01	Up
907	1.9E-01	5.8E-01	Up
906	2.0E-01	6.0E-01	Up
545	2.1E-01	6.2E-01	Up
566	2.3E-01	6.5E-01	Up
588	2.3E-01	6.6E-01	Up
1740	2.3E-01	6.6E-01	Up
644	2.4E-01	6.6E-01	Up
2210	2.4E-01	6.6E-01	Up
518	2.5E-01	6.6E-01	Up
1889	2.5E-01	6.6E-01	Up
710	2.7E-01	7.0E-01	Up
611	2.7E-01	7.0E-01	Up
582	2.8E-01	7.0E-01	Up
711	2.8E-01	7.0E-01	Up

Supplementary Table S5. Metabolomic pathway analysis of third trimester in future term Preeclampsia

Pathway Name	Match Status*	p-value	FDR^	Impact
Pyruvate metabolism	5/32	0.02175	0.51768	0.42654
Amino sugar and nucleotide sugar metabolism	2/88	0.045626	0.51768	0.0
Glyoxylate and dicarboxylate metabolism	4/50	0.050996	0.51768	0.14685
Glycerophospholipid metabolism	3/39	0.05735	0.51768	0.1249
Citrate cycle (TCA cycle)	3/20	0.063742	0.51768	0.16797
Galactose metabolism	3/41	0.092748	0.51768	0.22669
Arachidonic acid metabolism	1/62	0.096077	0.51768	0.0
Linoleic acid metabolism	1/15	0.096077	0.51768	0.0
alpha-Linolenic acid metabolism	1/29	0.096077	0.51768	0.0
Selenoamino acid metabolism	2/22	0.099305	0.51768	0.00321
Sphingolipid metabolism	2/25	0.11368	0.51768	0.00954
Glycerolipid metabolism	2/32	0.11504	0.51768	0.2283
Glycolysis or Gluconeogenesis	5/31	0.15411	0.53859	0.09576
Tyrosine metabolism	6/76	0.16668	0.53859	0.18202
Sulfur metabolism	2/18	0.16886	0.53859	0.03307
Taurine and hypotaurine metabolism	4/20	0.18707	0.53859	0.38489
Tryptophan metabolism	3/79	0.1977	0.53859	0.20083

*Significant metabolites ($p<0.05$) / Total metabolites in given pathway
^FDR: False Discovery Rate

Supplementary Table S6. Threshold values for each predictive logistic regression models

Predictors	AUC (95% CI)	Threshold*
**FIRST TRIMESTER MODELS**		
Parity, MAP (12wks), BMI (12wks)	0.565 (0.442-0.688)	0.32
Putrescine, Urea, Carnitine	0.701 (0.589-0.814)	0.40
TNF-α, RPL41, ATP5E, TBP	0.694 (0.578-0.811)	0.37
TNF-α, RPL41, ATP5E, TBP, Putrescine, Urea, Carnitine	0.745 (0.638-0.852)	0.32
**THIRD TRIMESTER MODELS**		
Parity, MAP (32wks), BMI (32wks)	0.525 (0.405-0.644)	0.35
Methylhistidine, Serotonin, Citrate, Hexose, Propylene glycol	0.761 (0.648-0.875)	0.45
GTPBP3, HLA-DR β-1 MHC	0.985 (0.956-1.000)	0.06
TEX15, SCG10	0.937 (0.862-1.000)	0.04
GTPBP3, HLA-DR β-1 MHC, MAP (32wks), BMI (32wks)	0.941 (0.879-1.000)	0.31
**SERIAL MODELS**		
MAP (32wks), MAP (12wks), BMI (12wks), BMI (32wks),	0.582 (0.460-0.705)	0.40
TEX15 (3rd tr), TBP (1st tr)	0.987 (0.961-1.000)	0.06
GTPBP3 (3rd tr), RPL41 (1st tr)	0.983 (0.953-1.000)	0.23
GTPBP3 (3rd tr), SCG10 (3rd tr), ATP5E (1st tr), BMI32wks, MAP32wks, MAP12wks	0.977 (0.949-1.000)	0.15
Urea (1st), SM C18:1 (1st), Citrate (3rd), Hexose (3rd),	0.817 (0.732-0.902)	0.31
Urea (1st), Hexose (3rd), SM C18:1 (1st), Citrate (3rd), MAP(32wks), BMI (12wks)	0.805 (0.717-0.894)	0.29

*Threshold is determined by Youden approach, which is the optimal cut-off for the logistic regression equation that maximizes the distance of the ROC to the diagonal line.

Supplementary Figure S1. Targeted third trimester proteomics for tPE prediction

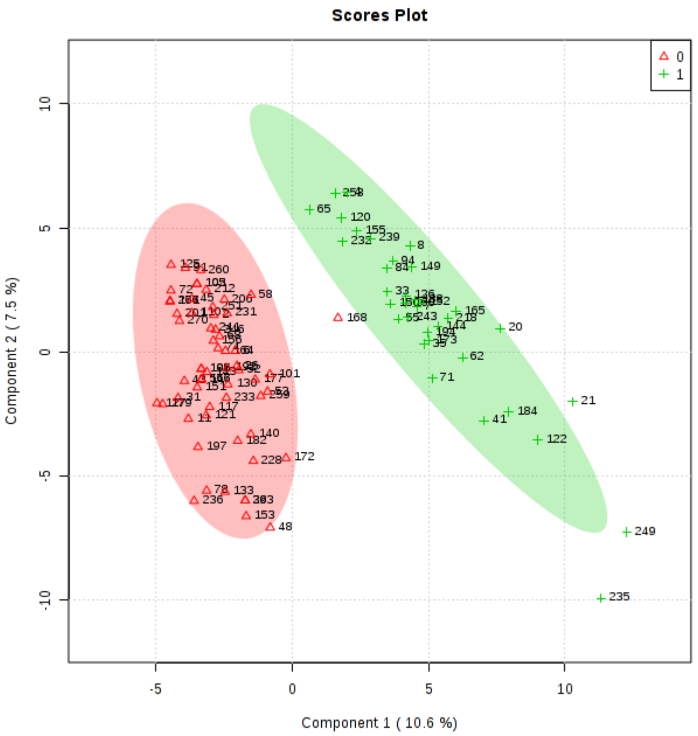

(A) 2 Dimensional PLS-DA plot

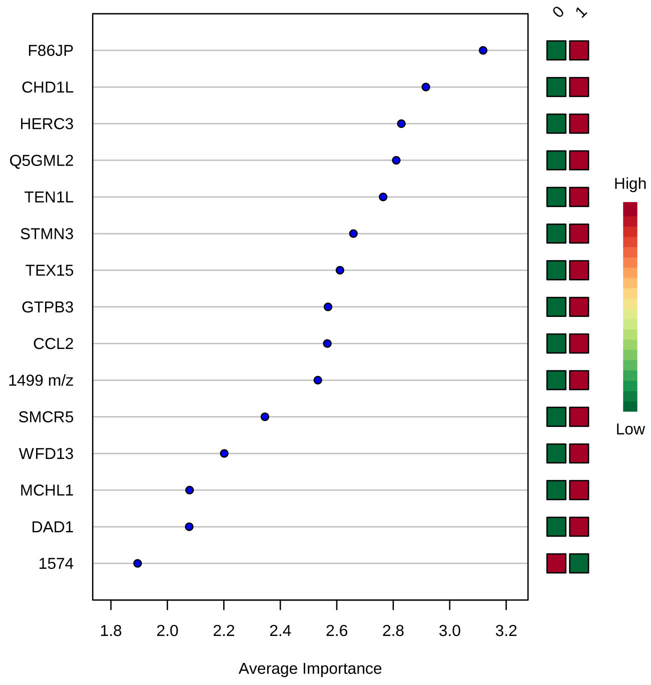

(B) VIP plot _ third trimester

The higher the VIP value (X-axis) the more discriminating the peptide.

Supplementary Methods Section

Metabolomic analysis

NMR-based metabolomic analysis

NMR spectra was acquired as described by Mercier et al., [1]. In brief, ^{1}H-NMR spectra were recorded at 300K on a 600-MHz Avance III HD Bruker spectrometer (Bruker Biospin Inc, Billerica, MA) equipped with a triple resonance inverse detection TCI cryoprobe operating at 600.13 MHz. In a randomized order, all spectra were acquired under automation using a previously reported pulse sequence [1]. Throughout the sequence all samples were kept at 4°C and heated to 25°C for 3 minutes prior to analysis. Two hundred and fifty six transients were acquired for each spectrum. The singlet produced by the DSS methyl groups was used as an internal standard for chemical shift referencing (set to 0 ppm, concentration 1000 μM) and for quantification all ^{1}H-NMR spectra were processed and analyzed using the Bayesil Software package [2].

DI-LC-MS/MS based targeted metabolomic analysis

Absolute IDQ kit – P180 (Biocrates Life Sciences AG, Innsbruk, Austria) with a TQ-S mass spectrometer coupled to an Acquity I Class ultra-pressure liquid chromatography (UPLC) system (Waters Technologies Corporation, Milford, MA, USA) was used to perform targeted analysis of metabolites including amino acids, acylcarnitines, biogenic amines, glycerophospholipids, sphingolipids, and sugars. AboluteIDQ manual protocol was used to analyze serum samples [3]. Briefly, serum samples were thawed on ice, vortexed and centrifuged at 4° C for 5 minutes at 2750 g. Ten microliters of blank, 3 zero samples, 7 calibration standards and 3 quality control samples were loaded onto the filters in the upper 96 well plate and then dried in nitrogen stream with 96 well plate positive pressure processor (Waters Technologies

Corporation, Milford, MA, USA). Subsequently 50 μl of phenylisothiocyanate derivatization solution was added to each well and left at room temperature for 20 minutes. The plate was dried again under nitrogen stream for 60 minutes, followed by addition of 300 μl of methanol containing 5 mM ammonium acetate for extraction of metabolites and shaking for 30 minutes. The extracts were filtered to the lower plate using nitrogen air flow in the positive pressure processor. The eluates were diluted with water for analysis of metabolites with the workflow using UPLC-MS and diluted with running solvent for flow injection analysis (FIA) – MS. Sample registration, automated calculation of metabolite concentrations and export of data were carried out with Biocrates MetIDQ software.

REFERENCES

1 Mercier, P., Lewis, M., Chang, D., Baker, D. & Wishart, D. Towards automatic metabolomic profiling of high-resolution one-dimensional proton NMR spectra. *J Biomol NMR* **49**, 307-323, doi:10.1007/s10858-011-9480-x (2011).

2 Ravanbakhsh, S. *et al.* Accurate, fully-automated NMR spectral profiling for metabolomics. *PLoS One* **10**, e0124219, doi:10.1371/journal.pone.0124219 (2015).

3 Bahado-Singh, R. O. *et al.* Serum metabolomic markers for traumatic brain injury: a mouse model. *Metabolomics* **12**, 100, doi:10.1007/s11306-016-1044-3 (2016).
